# Enhancement of spin-wave nonreciprocity in magnonic crystals via synthetic antiferromagnetic coupling

**DOI:** 10.1038/srep10153

**Published:** 2015-05-07

**Authors:** K. Di, S. X. Feng, S. N. Piramanayagam, V. L. Zhang, H. S. Lim, S. C. Ng, M. H. Kuok

**Affiliations:** 1 Department of Physics, National University of Singapore, Singapore 117551; 2 School of Physical and Mathematical Sciences, Nanyang Technological University, Singapore 637371

## Abstract

Spin-wave nonreciprocity arising from dipole-dipole interaction is insignificant for magnon wavelengths in the sub-100 nm range. Our micromagnetic simulations reveal that for the nanoscale magnonic crystals studied, such nonreciprocity can be greatly enhanced via synthetic antiferromagnetic coupling. The nonreciprocity is manifested as highly asymmetric magnon dispersion curves of the magnonic crystals. Furthermore, based on the study of the dependence of the nonreciprocity on an applied magnetic field, the antiparallel alignment of the magnetizations is shown to be responsible for the enhancement. Our findings would be useful for magnonic and spintronics applications.

The nonreciprocal propagation of waves is an interesting and important phenomenon with wide technological applications in signal processing and computing based on waves such as light, microwaves or spin waves (SWs).^1^,^2^,^3^,^4^,^5^,^6^,^7^,^8^ Wave nonreciprocity can exist in structures that are both asymmetric and nonlinear,^9^ or systems with broken time-reversal symmetry.^7^ For application purposes, nonreciprocity is essential for unidirectional wave propagation and suppression of cross-interference between devices within a circuit.

It is well known that nonreciprocity of SWs can arise from the presence of classical dipole-dipole interaction in asymmetric structures.^4^,^6^,^8^,^10^ For instance, nonreciprocal microwave devices such as isolators and circulators extensively rely on nonreciprocal magnetostatic surface waves in ferrites. Nonreciprocal devices are also important for the stabilization of future integrated magnonic circuits, in which SWs serve as information carriers. However, for wavelengths in the sub-100nm range, the spin-wave nonreciprocity induced by dipolar interaction is generally weak, and therefore not a significant factor for device miniaturization. To realize stronger nonreciprocity at this scale, one can resort to physical mechanisms other than the dipolar interaction, such as spin-wave nonreciprocity induced by a symmetry-breaking magnetic field.^7^ Another promising method is via the chiral Dzyaloshinskii–Moriya interaction, which has been demonstrated to induce very strong spin-wave nonreciprocity.^3^,^11^,^12^,^13^,^14^

Magnonic crystals (MCs) are artificial crystals with periodically modulated magnetic and structural properties. Exhibiting band gaps in which spin-wave propagation is prohibited, MCs can be used to manipulate the propagation of SWs. Here, instead of seeking a different mechanism, we propose an alternative method to enhance the dipolar-interaction-induced spin-wave nonreciprocity in MCs via synthetic antiferromagnetic (AFM) coupling, which changes the symmetry property of dipolar interaction. In the absence of the AFM coupling, the nonreciprocity is found to be negligible. By studying the magnetic field dependence of the nonreciprocity, we found that the enhancement is closely correlated with the AFM alignment of the magnetizations. Finally, a modification of the AFM MCs by magnetic anisotropy is proposed to facilitate miniaturization of devices based on them.

## Results

### Magnonic crystals with respective FM and AFM couplings

**Figure 1:**
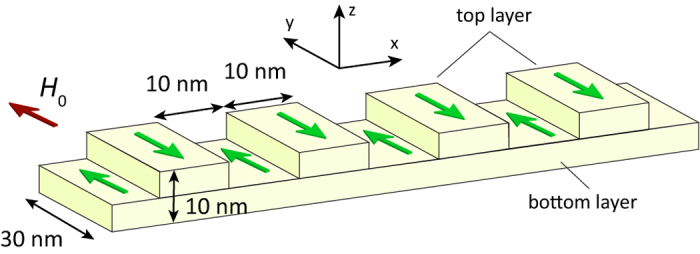
Schematic diagram of the magnonic crystal with AFM coupling. The external magnetic field *H*_0_ is applied transverse (along *y*-axis) to the long axis of the nanostripe. Green arrows represent the respective directions of the magnetizations of the top and bottom layers under an external field *H*_0_ = 0.1 T/μ_0_ along the *y*-axis.

The MC considered is a 2μm-long, 30nm-wide ferromagnetic nanostripe decorated with a periodic array of nanocuboids, as shown in Fig. 1.^15^,^16^,^17^ The crystal has a lattice constant *a* = 20 nm and consists of two layers of equal thickness, between which the exchange coupling can be of either the AFM or ferromagnetic (FM) type. As is usually done in recording media applications,^18^,^19^,^20^ the AFM interlayer coupling can be realized by separating the two FM layers by a nonmagnetic (e.g. ruthenium) spacer layer, which could be thinner than 1 nm, and as such has been neglected in our study.^21^,^22^ For simplicity, we assume that the MC is made of only one material (Permalloy), with a saturation magnetization *M*_S_ = 800 kA/m, and an exchange constant *A*_0_ = 13 pJ/m. The AFM coupling between the two layers is taken to be *A*_1_ = −3 pJ/m. An external magnetic field *H*_0_ is applied transverse, in the y-direction, to the long axis of the crystal.

### Band structures

**Figure 2:**
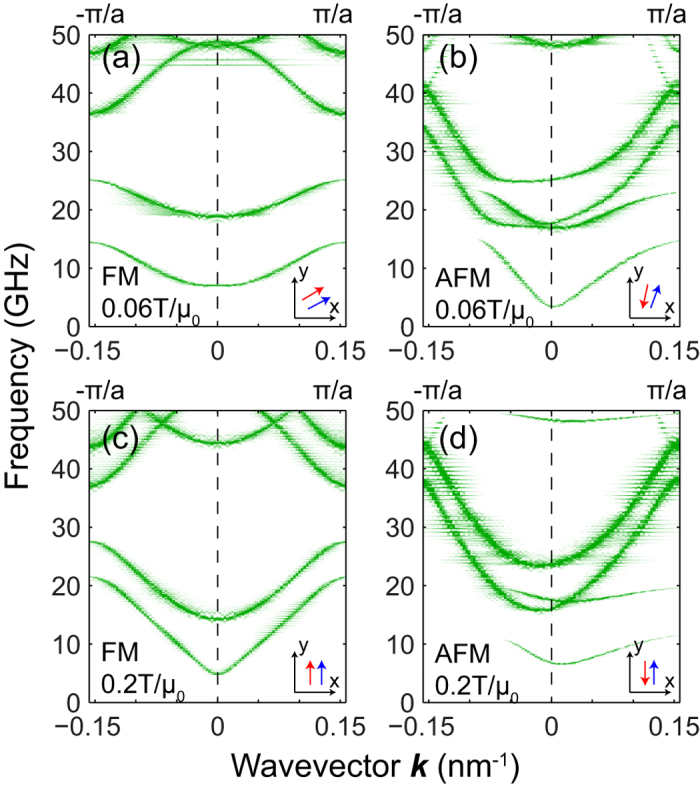
Calculated dispersion curves of the MCs (*a* = 20 nm) with (**a**, **c**) FM and (**b**, **d**) AFM coupling under transversely applied fields *H*_0_ = 0.06 and 0.2 T/μ_0_. Insets: Corresponding directions of the equilibrium magnetizations of the top (red arrows) and bottom (blue arrows) layers.

Micromagnetic simulations were adopted to investigate the SWs dispersion relations in the MCs (See Methods section for details). Figure 2 shows the calculated dispersion relations in the first Brillouin zone (BZ) of the MCs with respective FM and AFM couplings under applied transverse fields of 0.06 and 0.2 T/μ_0_. It is obvious that the dispersion curves of the MC with FM coupling are almost symmetric with respect to the spin-wave wavevector *k*. This is consistent with the argument that since the spin-wave wavelengths studied are in the sub-100nm range, nonreciprocity is expected to be insignificant. To rely on such a weak nonreciprocity in MC with FM coupling, devices based on it necessarily have to have very large footprints. In contrast, the AFM-coupled MC possesses highly asymmetric dispersion curves. It is obvious that for the same wavevector, counter-propagating SWs exhibit very different group velocities and frequencies, and generally, the AFM MC has higher group velocities than those of the FM MC. Both crystals exhibit a few band gap openings due to Bragg scattering in the periodic structures. It is interesting that the band gap openings of the AFM MC are not at the BZ boundaries anymore, because the dispersion curves of counter-propagating waves no longer intersect at the BZ boundary due to their different slopes.^5^ Note that this property is a natural property of gyrotropic periodic materials.^23^,^24^ Another interesting property of the band structure of the MC with AFM coupling is that the SWs now acquire nonzero group velocities when *k* = 0.

**Figure 3:**
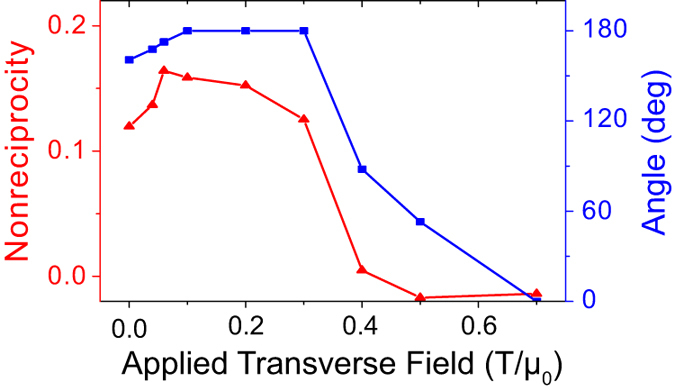
The calculated (**a**) nonreciprocity (denoted by red triangles) and (**b**) angle (denoted by blue squares) between the respective magnetizations of the top and bottom layers as functions of applied transverse field of the AFM crystal (*a* = 20 nm). The solid lines only serve as a guide to the eye.

We next study the dependence of the spin-wave nonreciprocity on the applied transverse field *H*_0_. For simplicity, nonreciprocity is defined as 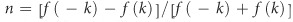, where *f* (*k*) is the frequency of the lowest magnon branch at *k* = 0.05 nm^−1^. Figure 3 shows that nonreciprocity is most pronounced for 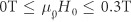 approximately, and is insignificant for 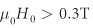. This can be qualitatively explained by the different relative orientations of the equilibrium magnetizations of the top and bottom layers under different external fields. For 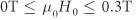, the angle between the two magnetizations is nearly 180°, i.e. the magnetizations are almost perpendicular to the *x*-axis, and the counter-propagating surface waves are subjected to highly asymmetric effective fields. For 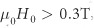, the angle gradually decreases with increasing transverse field, resulting in the lowering of the asymmetry of the effective field, and thus the nonreciprocity. Hence, the enhanced nonreciprocity is a consequence of the antiparallel alignment of the respective magnetizations of the top and bottom layers of the MC. Interestingly, our calculation shows that the spin-wave nonreciprocity (*H*_0_ = 0.1 T) is dependent on the interlayer AFM exchange coupling parameter, with a maximum value obtained at *A*_1_ ≈ –1 pJ/m.

**Figure 4:**
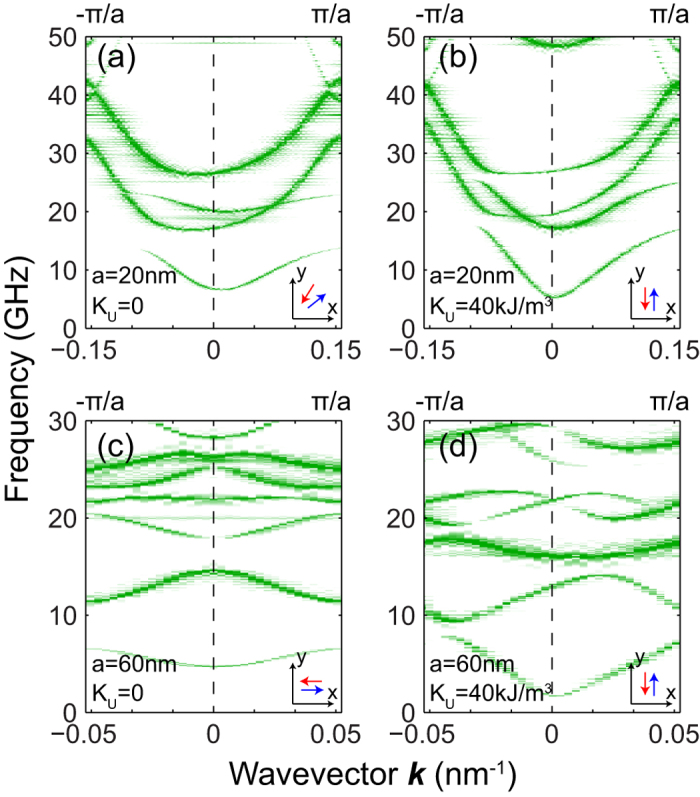
Band structures, calculated for zero applied field, of various AFM magnonic crystals for (**a**) lattice constant *a* = 20 nm, magnetic anisotropy *K*_U_ = 0 kJ/m^3^, (**b**) *a* = 20 nm, *K*_U_ = 40 kJ/m^3^, (**c**) *a* = 60 nm, *K*_U_ = 0 kJ/m^3^ and (**d**) *a* = 60 nm, *K*_U_ = 40 kJ/m^3^. Insets show the corresponding directions of the equilibrium magnetizations on the top (red arrows) and bottom (blue arrows) layers of the samples.

It is noteworthy that the nonreciprocity of the AFM MC is significant even in the absence of an applied field. This is because, under zero field, the equilibrium magnetizations of the crystal are oriented at a large angle to the long crystal axis [see inset of Fig. 4(a)], due to the shape anisotropy. Another AFM MC with the same magnetic parameters but a larger lattice period of *a* = 60 nm was also studied. As shown in Fig. 4(c), its band structure is symmetric, indicating an absence of nonreciprocity. This is because the equilibrium magnetizations, in zero external field, are either parallel or antiparallel to the MC’s long axis and the wavevector. Hence, we deduce that the AFM configuration of the magnetizations is a necessary but not sufficient condition for enhanced nonreciprocity. To achieve nonreciprocity in this crystal, an external transverse field should be applied to favor the propagation of surface waves. This is consistent with the finding of Ref. 4, that a non-zero out-of-plane component (*y* direction in this paper) of the static magnetization is necessary for appearance of nonreciprocity.

Devices based on MCs that rely on the application of a magnetic field for their operation are necessarily bulkier than those that do not, and do not allow for easy integration into integrated magnonic circuits. To overcome this problem, the magnetization of the bottom layer can be pinned along the *y*-direction (perpendicular to the long axis of the crystal) by either suitable magnetocrystalline anisotropy, or effective surface pinning through exchange bias^25^ by another contacting antiferromagnetic layer. In the following simulations, we assume that the bottom layers of the *a* = 20 nm and *a* = 60 nm MCs have modest second-order uniaxial magnetic anisotropies. The anisotropies have easy axes along the *y*-direction and an anisotropy constant *K*_U_=40 kJ/m^3^. In the absence of an external magnetic field, the equilibrium magnetizations of the bottom layers of both crystals are aligned along the *y*-direction. The simulated magnon dispersions are presented in Fig. 4. Figure 4(b) shows the highly asymmetric nature of dispersion of the *a* = 20 nm MC even for *H*_0_ = 0. Under zero external field, its nonreciprocity is higher than that of the MC with *K*_U_ = 0 [see Fig. 4(a)]. Interestingly, the *a* = 60 nm MC has drastically different dispersion relations [see Fig. 4(c,d)] depending on the value of *K*_U_. Even in the absence of an applied field, its dispersion is highly asymmetric for *K*_U_=40 kJ/m^3^. As external fields are unnecessary, this effect makes possible the on-chip integration of magnonic devices based on such MCs.

## Discussion

**Figure 5:**
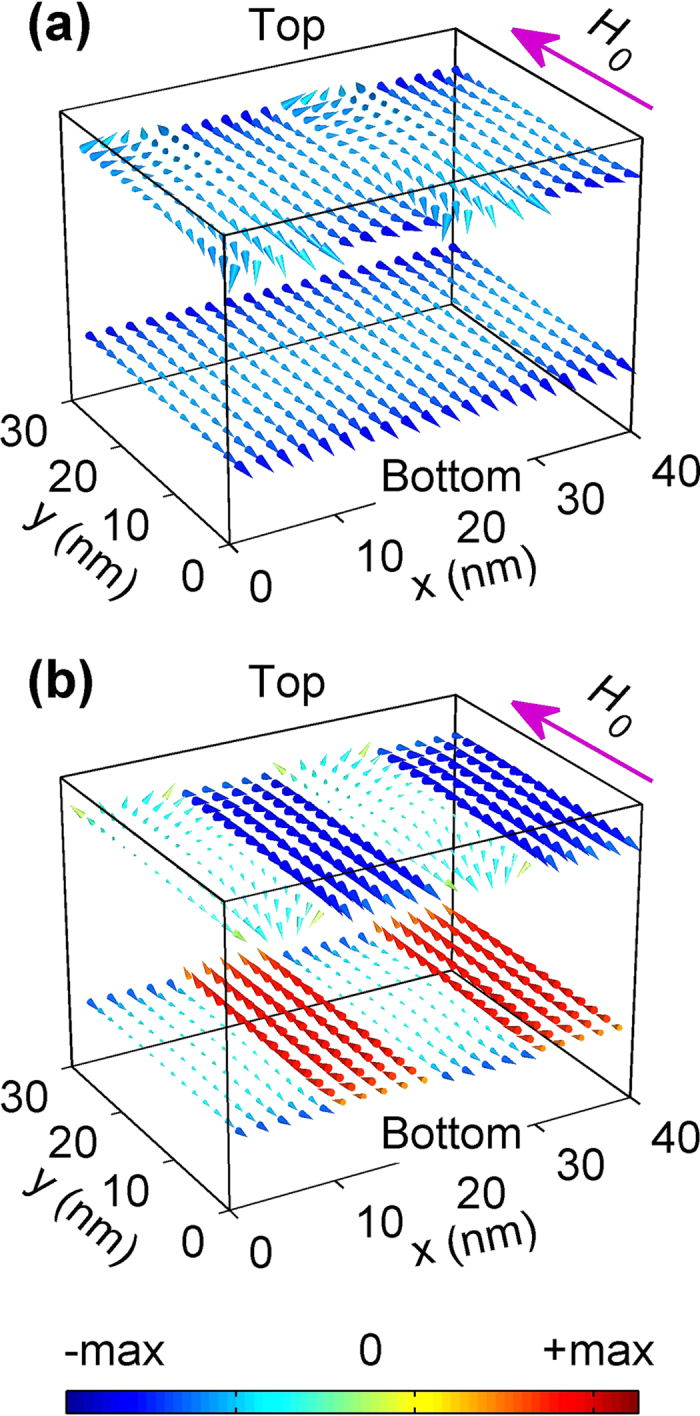
The simulated effective internal field (indicated by cones) 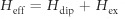 within two unit cells of the *a* = 20 nm magnonic crystals in an external field *H*_0_ = 0.2 T/μ_0_. Magnonic crystal with (**a**) FM and (**b**) AFM interlayer-coupling. The magnitude and *y*-component of *H*_eff_ are represented by the size and color of the cones (see bottom color bar), respectively.

The enhanced nonreciprocity is a result of the large contrast in the respective magnetizations and the effective fields of the top and bottom layers of the MCs. Depending on their propagation direction, SWs in the Damon–Eschbach geometry tend to localize on either the top or bottom surface of the MC. Figure 5 shows the distribution of the internal field 
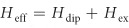
 in the top and bottom layers, where *H*_dip_ and *H*_ex_ are the effective dipolar and exchange fields, respectively. It is evident that the distribution of the internal field of the AFM MC exhibits a larger contrast between the two layers than that of the FM crystal. Therefore, because of the nonreciprocal localization of counter-propagating SWs, they experience very different environments, thus resulting in their asymmetric frequencies. It is noteworthy that the observed nonreciprocity has its origin in the dipole-dipole interaction. If the demagnetizing field is switched off in the simulations, all the simulated dispersion curves are symmetric, irrespective of the applied external field and the type of coupling, i.e. whether FM or AFM, between the top and bottom layers. Our result is consistent with that of Ref. 4, that is, SWs in dipolarly-coupled magnetic nanopillar arrays can be nonreciprocal, which could be enhanced for AFM ground states.

In summary, we have proposed an approach whereby the weak spin-wave nonreciprocity of an MC, for spin-wave wavelengths in the sub-100 nm range, can be greatly enhanced via synthetic AFM coupling. Our micromagnetic simulations show that the dispersion curves of such an MC will be modified, by the synthetic AFM coupling, to become highly asymmetric with respect to counter-propagating spin-waves. The calculated magnetic-field dependence of the nonreciprocity reveals that the antiparallel alignment of magnetizations of the top and bottom layers of the MC is responsible for the enhanced nonreciprocity. Finally, we demonstrated that the presence of magnetic anisotropy in the crystal can lead to spin-wave nonreciprocity even in zero applied magnetic field. Our findings could be used to design the building blocks, e.g. isolators and circulators, for information processing based on SWs and microwaves.

## Methods

### Micromagnetic simulation

Micromagnetic simulations were performed by solving the Landau-Lifshitz-Gilbert equation^26^ 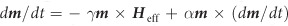 implemented in the OOMMF package.^27^ The exchange energy density at the computational cell *i* takes the form 
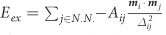
 where the summation is over nearest neighbors, 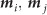 are the unit vectors along magnetization at cell *i* and *j*, 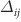 is the step size between cell *i* and *j*, and 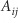 is the exchange parameter between cell *i* and *j*. For intralayer directions (*x-y* plane), 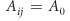 while for interlayer directions (*z* direction), 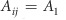 when the coupling is of AFM type and 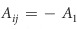 when FM type. The dimensionless Gilbert damping constant *α* was set to 1 × 10^−4^, and the crystals were discretized to computation cells of mesh dimensions Δ*x*Δ*y*Δ*z* = 2 × 2 × 5 nm^3^. The MCs were first relaxed with an applied magnetic field to obtain equilibrium magnetizations. An oscillating excitation field, 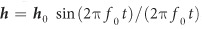, where *f*_0_ = 100 GHz, was applied to the central portion of the crystals to excite SWs propagating in the –*x* and +*x* directions.^28^ Their dispersion relations were then calculated by performing Fourier transforms of the obtained dynamic magnetizations in both space and time.

## Author Contributions

S.N.P. and H.S.L. conceptualized the project. K.D. and S.X.F. performed the calculations. H.S.L. supervised the project. V.L.Z., S.C.N. and M.H.K. participated in the analysis and interpretation of the data. All authors discussed and commented the manuscript.

## Additional Information

**How to cite this article**: Di, K. *et al*. Enhancement of spin-wave nonreciprocity in magnonic crystals via synthetic antiferromagnetic coupling. *Sci. Rep.* **5**, 10153; doi: 10.1038/srep10153 (2015).
